# Influence of white blood cell count trajectories on the risk of differentiation syndrome during induction therapy with all-trans-retinoic acid and arsenic trioxide in pediatric acute promyelocytic leukemia

**DOI:** 10.3389/fped.2025.1742083

**Published:** 2026-01-07

**Authors:** Houxin Fu, Yuchen Liu, Shaoyan Hu

**Affiliations:** 1 Department of Hematology and Oncology, Children’s Hospital of Soochow University, Suzhou, China; 2 Department of Neurosurgery, Children's Hospital of Soochow University, Suzhou, China; 3 Jiangsu Pediatric Hematology and Oncology Center, Suzhou, China; 4 Pediatric Hematology & Oncology Key Laboratory of Higher Education Institutions in Jiangsu Province, Suzhou, China

**Keywords:** acute promyelocytic leukemia, all-trans-retinoic acid, arsenic trioxide, differentiation syndrome, latent growth mixture modeling, white blood cell count

## Abstract

**Objective:**

This study aims to investigate the association between early dynamic trajectories of white blood cell (WBC) count and the risk of differentiation syndrome (DS) during induction therapy with All-trans-retinoic Acid (ATRA) combined with arsenic trioxide (ATO) in pediatric patients with acute promyelocytic leukemia (APL).

**Methods:**

A retrospective cohort of pediatric APL patients treated with ATRA and ATO induction therapy between January 2016 and December 2024 is analyzed. Latent growth mixture modeling (LGMM) is employed to identify distinct WBC count trajectories over the first seven days of induction therapy. DS is diagnosed according to Frankel's criteria. Logistic regression analyses are performed to evaluate associations between WBC trajectory classes and the occurrence of DS, treatment-related complications, and transfusion requirements.

**Results:**

A total of 93 patients are included, with an overall incidence of DS of 40.9% during induction therapy. Four distinct WBC trajectory classes are identified: Class 1 (high-level increasing group), Class 2 (high-level decreasing group), Class 3 (persistently low-level group), and Class 4 (low-level increasing group). After adjustment for potential confounders, patients in Class 1 (OR: 11.37, 95% CI: 1.17–124.71) and Class 4 (OR: 8.34, 95% CI: 1.94–35.92) remain at significantly increased risk of DS compared to those in Class 3, while no significant difference in DS risk is observed between Class 2 and Class 3. Furthermore, patients in Class 1 require more frequent transfusion support, including red blood cells, platelets, and plasma (*p* < 0.001) during induction therapy.

**Conclusion:**

The trajectory of WBC count during ATRA and ATO induction therapy may serve as an indicator for predicting the risk of DS in pediatric APL patients.

## Introduction

Acute promyelocytic leukemia (APL) represents a distinct subtype of acute myeloid leukemia (AML), accounting for approximately 10%–15% of AML cases. It is characterized by abnormal white blood cell (WBC) counts, thrombocytopenia, coagulopathy, and a high risk of hemorrhage (1–4). The advent of combination therapy with All-trans-retinoic Acid (ATRA) and arsenic agents has markedly improved the cure rate for patients with APL (5, 6). Differentiation syndrome (DS) is a common complication associated with treatment using ATRA and arsenic compounds such as arsenic trioxide (ATO), with reported incidence rates as high as 48% (7–9). DS may lead to severe, potentially life-threatening complications; thus, early recognition and prompt intervention are critical (10, 11).

Recent studies identify leukocytosis and WBC doubling during ATRA-ATO induction therapy as independent risk factors for DS in patients with APL (1, 12). However, WBC counts fluctuate dynamically during induction therapy, and to date, no studies have systematically examined how different WBC trajectory patterns influence the risk of DS.

This study aims to identify early WBC trajectory subtypes during ATRA-ATO induction therapy in pediatric APL and to evaluate the associations between these trajectory patterns and the risk of DS, as well as other treatment-related adverse events. The goal is to establish a more refined risk stratification model that facilitates early identification of high-risk patients and informs optimized intervention strategies, thereby improving clinical outcomes.

## Methods

### Study patient cohorts

**Figure 1:**
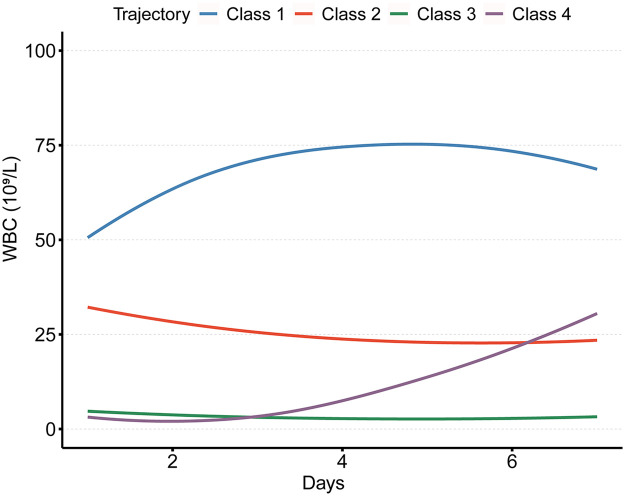
Flowchart for study participants enrolling.

In this retrospective study, 93 pediatric patients diagnosed with APL were treated with a combination of ATRA-ATO therapy at the Department of Hematology, Children's Hospital of Soochow University, between 2016 and 2024. The inclusion criteria are as follows: (1) diagnosis of APL confirmed by detection of the PML-RAR*α* rearrangement using reverse transcription-polymerase chain reaction (RT-PCR) (13); (2) age ≤18 years; and (3) initiation of ATRA-ATO therapy immediately after diagnosis. Patients lacking data on peripheral WBC counts are excluded from the study (Figure 1).

Treatment for all patients follows the NCCN guidelines (14), primarily involving a combination of ATRA-ATO therapy. Upon confirmation of APL, therapy was initiated immediately: ATRA 25 mg/(m^2^·day) plus ATO 0.15 mg/(kg·day) administered continuously until complete remission (CR) was achieved. Therapeutic-dose low molecular weight heparin (LMWH) was initiated concomitantly with ATRA–ATO and continued until coagulopathy resolved. Upon diagnosis of DS during induction therapy for newly diagnosed APL, ATRA is immediately reduced or discontinued, and dexamethasone, along with appropriate supportive care, is administered until resolution of clinical symptoms.

The study was approved by the Ethics Committee of the Children's Hospital of Soochow University(2021KS003). Given that no additional interventions were conducted, the requirement for informed consent was waived by the institutional review board. All data utilized were anonymized and could not be traced back to individual patients.

### Exposure

In this study, exposure is defined as the trajectory of WBC count variations from day 1 to day 7 following the initiation of ATRA-ATO therapy. The identification of distinct WBC count trajectories was performed using Latent Growth Mixture Modeling (LGMM). An unconditional quadratic growth model was specified within the LGMM framework to model the WBC count from day 1 to day 7 of induction therapy. To determine the optimal number of latent classes, a series of models was estimated, beginning with a single-class quadratic growth model and incrementally increasing the number of classes up to four. Model fit was compared using the following statistical indices: log-likelihood, Akaike Information Criterion (AIC), Bayesian Information Criterion (BIC), sample-size adjusted BIC (SABIC), and entropy. Lower values of AIC, BIC, and SABIC, along with higher log-likelihood and entropy, signify a better model fit. The model estimation utilized robust maximum likelihood estimation, and all models successfully converged based on standard criteria (15).

### Data collection

Baseline data include demographic characteristics (age, sex, height, and weight), FLT3-ITD mutation status, presence of infection at initial diagnosis, disseminated intravascular coagulation (DIC), ultrasound findings (hepatosplenomegaly), bone marrow promyelocyte percentage [BM promyelocytes (%)], CD117 expression by flow cytometry, CD34 expression by flow cytometry, baseline WBC count, hemoglobin (HB), platelet count, absolute neutrophil count, absolute monocyte count, serum creatinine (SCr), blood urea nitrogen (BUN), international normalized ratio (INR), D-dimer, fibrinogen (FIB), albumin, lactate dehydrogenase (LDH), creatine kinase-MB (CKMB), and cardiac troponin T (cTNT). Baseline WBC is defined as the highest value recorded before the initiation of retinoic acid therapy, or, if unavailable, as the first WBC measurement upon hospital admission. Missing covariate data are addressed through multiple imputation. To reduce information bias, variables with a missing rate exceeding 10% are excluded from the analysis.

### Outcome

The primary outcome of this study is the occurrence of DS in pediatric patients with APL during treatment with ATRA-ATO in combination with arsenic. The diagnosis of DS is based on the Frankel criteria (3), which include unexplained fever, dyspnea, pleural or pericardial effusion, pulmonary infiltrates, renal failure, hypotension, and weight gain ≥5 kg. A diagnosis of DS is established when at least two of these manifestations are present. Each potential event was independently reviewed by two senior hematologists; disagreements were resolved by consensus or a third senior physician, ensuring objective and consistent outcome assessment. Only DS episodes occurring during the ATRA-ATO induction period were counted.

Secondary outcomes include the development of various treatment-related complications, such as abnormal liver function and infections, as well as transfusion requirements, including platelet, red blood cell, and plasma transfusion volumes.

### Statistical analysis

All statistical analyses are performed using R software (version 4.4.2) and Python software (version 3.13). LGMM was conducted using the lcmm package (version 2.1.0) in R. Continuous variables with a normal distribution are described using means and standard deviations, and differences across trajectory classes are assessed using analysis of variance (ANOVA). Variables not normally distributed are summarized as medians with interquartile ranges (IQRs), and intergroup differences are evaluated using the Kruskal–Wallis test. Categorical variables are presented as frequencies and percentages, and differences between groups are examined using the *χ*^2^ test or Fisher's exact test, as appropriate. Odds ratios (ORs) and 95% confidence intervals (CIs) are estimated using univariable and multivariable logistic regression analyses. A two-sided *p*-value of less than 0.05 is considered statistically significant.

## Results

### LGMM analysis and baseline characteristics

The goodness-of-fit statistics for the LGMM models are presented in

Table 1. The four-class model yields the lowest values for the AIC, BIC, and SABIC, indicating the best model fit. Therefore, the four-class model is selected as the optimal solution.

**Table 1:** Statistics for choosing the best number of classes.

Number of classes	Log likelihood	AIC	BIC	SABIC	Entropy	%class1	%class2	%class3	%class4
2.0	−1,876.9	3,785.8	3,826.3	3,775.8	0.9	36.6	63.4		
3.0	−1,871.0	3,781.9	3,832.6	3,769.4	0.9	35.5	3.2	61.3	
4.0	−1,857.9	3,763.9	3,824.7	3,748.9	0.9	7.5	21.5	57.0	14.0

AIC, akaike information criterion; BIC, Bayesian information criterion; SABIC, sample-adjusted information criterion.

**Figure 2:**
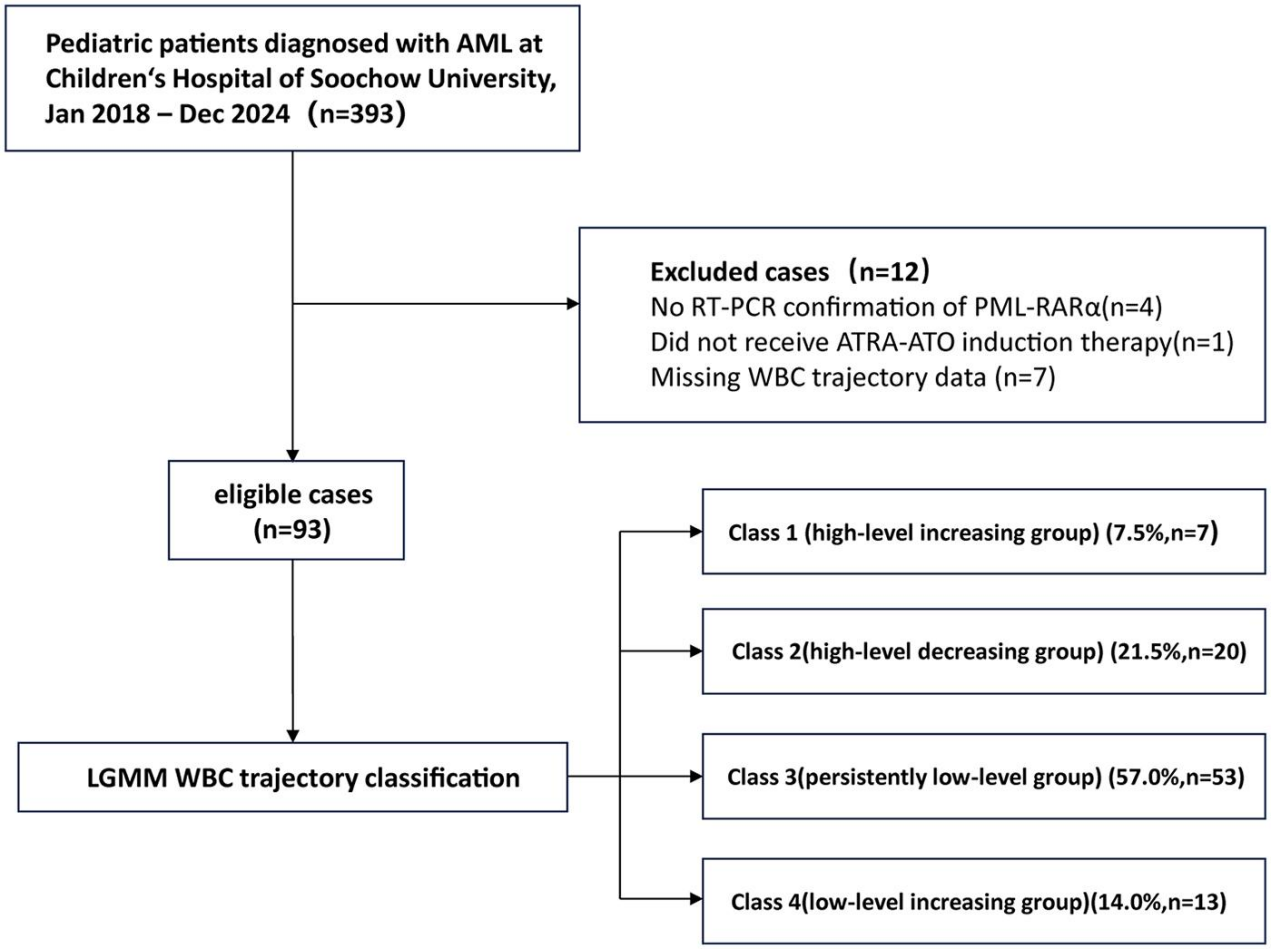
Four classes identified by trajectories of white blood cell count.

The trajectories of WBC count changes across the four-class LGMM model are depicted in Figure 2. Class 1 (high-level increasing group) comprises 7.5% of the cohort and is characterized by the highest initial WBC count, which rises before declining, presenting an inverted V-shaped pattern. Class 2(high-level decreasing group) includes 21.5% of patients and begins with moderately elevated WBC counts, lower than those in Class 1, followed by a gradual decrease, ultimately remaining higher than the levels in Class 3. Class 3(persistently low-level group) accounts for 57.0% of patients, with consistently low and stable WBC counts throughout the observation period. Class 4(low-level increasing group), representing 14.0% of the cohort, shows initially low and stable WBC levels, followed by a progressive increase, ultimately exceeding those in Class 2 but remaining below those in Class 1.

A total of 93 patients are included in the study, and their baseline characteristics are summarized in Table 2. The incidence of DS following treatment with ATRA-ATO in patients with APL is 40.9%. The median age of the patients is 8.3 years, and 55.9% are male. Compared with other trajectory classes, patients in Class 1 exhibit higher baseline neutrophil counts and INR values at diagnosis, a greater frequency of splenomegaly, and the highest incidence of DS (85.7%). Patients in Class 4 have the highest body mass index (BMI) and a DS incidence of 69.2%, second only to that of Class 1. Class 3 includes the largest number of patients and is characterized by the lowest BMI, INR, and DS incidence (22.6%).

**Table 2:** Baseline characteristics of four classes.

Characteristic	Total (*n* = 93)	Class 1 (*n* = 7)	Class 2 (*n* = 20)	Class 3 (*n* = 53)	Class 4 (*n* = 13)	*p*-value
Age(year)	8.3 (5.6, 11.2)	11.2 (7.9, 12.1)	7.6 (5.5, 12.5)	8.3 (5.5, 11.2)	10.2 (7.7, 10.9)	0.507
Female, *n* (%)	41 (44.1)	24 (45.3)	10 (50)	2 (28.6)	5 (38.5)	0.799
BMI	16.4 (15.4, 20.1)	18.1 (16.5, 19.6)	17.1 (15.1, 23.0)	16.0 (14.9, 18.6)	19.1 (17.3, 20.2)	0.035
FLT3-ITD, *n* (%)	29 (31.2)	4 (57.1)	7 (35)	16 (30.2)	2 (15.4)	0.31
Infection, *n* (%)	57 (61.3)	5 (71.4)	15 (75)	28 (52.8)	9 (69.2)	0.29
DIC, *n* (%)	56 (60.2)	6 (85.7)	16 (80)	27 (50.9)	7 (53.8)	0.067
Hepatomegaly, *n* (%)	29 (31.2)	4 (57.1)	6 (30)	14 (26.4)	5 (38.5)	0.359
Splenomegaly, *n* (%)	23 (24.7)	6 (85.7)	4 (20)	9 (17)	4 (30.8)	0.003
Hemoglobin(g/L)	84.0 (72.0, 95.0)	84.0 (71.5, 89.0)	76.5 (67.2, 91.0)	87.0 (74.0, 97.0)	83.0 (74.0, 97.0)	0.301
Neutrophil(10^9^ /L)	0.8 (0.3, 3.7)	25.6 (14.7, 109.6)	4.6 (2.3, 8.6)	0.4 (0.2, 1.0)	0.4 (0.3, 0.5)	< 0.001
Platelet(10^9^ /L)	36.0 (22.0, 80.0)	28.0 (18.5, 60.0)	28.5 (21.8, 55.0)	41.0 (27.0, 80.0)	82.0 (22.0, 87.0)	0.297
Bone marrow blasts (%)	87.0 (75.0, 91.0)	95.0 (82.0, 95.5)	87.9 (75.0, 92.9)	85.0 (74.0, 89.0)	87.0 (73.0, 89.0)	0.246
CD117 flow (%)	89.0 (16.9, 97.1)	11.5 (3.3, 57.2)	88.9 (48.3, 96.3)	89.1 (25.7, 97.8)	91.1 (16.5, 95.2)	0.546
CD34+, No. of pts (%)	46 (49.5)	2 (28.6)	8 (40)	30 (56.6)	6 (46.2)	0.383
DD, *n* (%)	52 (55.9)	6 (85.7)	12 (60)	25 (47.2)	9 (69.2)	0.174
INR, Mean ± SD	1.4 ± 0.3	1.6 ± 0.3	1.4 ± 0.2	1.3 ± 0.2	1.4 ± 0.3	0.003
FIB(g/L)	1.6 (1.2, 2.2)	1.2 (1.0, 1.5)	1.3 (0.9, 2.0)	1.8 (1.4, 2.3)	1.9 (1.2, 2.4)	0.104
ALB(g/L)	44.5 (41.6, 47.4)	44.7 (43.1, 45.0)	44.0 (39.1, 47.9)	44.0 (41.9, 47.0)	46.2 (43.1, 48.3)	0.776
BUN(mmol/L)	4.2 (3.5, 4.9)	4.9 (4.6, 5.3)	4.2 (3.2, 4.7)	4.1 (3.5, 4.8)	4.1 (3.5, 5.7)	0.185
Cr(µmol/L)	36.8 (30.1, 46.0)	46.0 (37.6, 48.6)	34.8 (31.7, 47.5)	34.5 (27.6, 44.6)	37.4 (36.8, 45.3)	0.106
cTnT(ng/L)	3.0 (2.4, 5.6)	0.0 (0.0, 0.2)	4.6 (2.6, 6.8)	2.6 (2.4, 5.3)	3.0 (2.6, 3.4)	0.067
CKMB(U/L)	0.9 (0.6, 1.8)	0.7 (0.6, 1.0)	0.7 (0.5, 1.1)	1.1 (0.6, 2.5)	0.7 (0.3, 1.6)	0.054
DS, *n* (%)	38 (40.9)	6 (85.7)	11 (55)	12 (22.6)	9 (69.2)	< 0.001

BMI, body mass index; DIC, disseminated intravascular coagulation; DD, D-dimer elevation; INR, international normalized ratio; FIB, fibrinogen; ALB, albumin; BUN, blood urea nitrogen; Cr, creatinine; cTnT, cardiac troponin T; CK-MB, creatine kinase-MB; DS, differentiation syndrome.

### Univariate and multivariate analyses

Univariate analysis demonstrated that, compared to Class 3, patients in Class 1 (OR: 20.5, 95% *CI*: 2.24–187.36), Class 2 (OR: 4.18, 95% *CI*: 1.40–12.43), and Class 4 (OR: 7.69, 95% *CI*: 2.01–29.42) were at significantly higher risk of developing DS. In addition, infection at diagnosis (OR: 2.51, 95% *CI*: 1.03–6.15), elevated INR (OR: 14.25, 95% *CI*: 2.23–90.96), and lower FIB levels (OR: 0.43, 95% *CI*: 0.23–0.80) are identified as predictors of DS. Multivariate logistic regression further confirms that patients in Class 1 (OR: 11.37, 95% *CI*: 1.17–124.71) and Class 4 (OR: 8.34, 95% *CI*: 1.94–35.92) remain at significantly increased risk for DS compared to those in Class 3, while no significant difference in DS risk is observed between Class 2 and Class 3. FIB is identified as an independent protective factor against the development of DS. The results of both univariate and multivariate analyses are presented in Table 3.

**Table 3:** Predictive factors for the development of DS during induction with all-­trans-­retinoic acid and arsenic trioxide in univariate and multivariate analysis.

Characteristic	Univariate analysis			Multivariate analysis		
	OR	95% CI	*p*-value	OR	95% CI	*p*-value
Class 1	20.5	2.24–187.36	0.007	12.07	1.17–124.71	0.037
Class 2	4.18	1.4–12.43	0.01	2.69	0.82–8.84	0.103
Class 3	Reference			Reference		
Class 4	7.69	2.01–29.42	0.003	8.35	1.94–35.92	0.004
Female, *n* (%)	0.87	0.38–2.01	0.749			
Age(year)	1.05	0.93–1.18	0.422			
BMI	1.09	0.98–1.21	0.119			
Infection	2.51	1.03–6.15	0.044	2.57	0.86–7.64	0.089
Bone marrow blasts (%)	1.02	1–1.05	0.109			
FLT3.ITD	2.35	0.96–5.75	0.061			
Hepatomegaly, *n* (%)	1.91	0.78–4.65	0.154			
Splenomegaly, *n* (%)	1.85	0.71–4.78	0.206			
DIC, *n* (%)	2.2	0.91–5.3	0.078			
CD117 flow (%)	1	0.99–1.01	0.594			
CD34, *n* (%)	0.5	0.22–1.17	0.111			
Hemoglobin(g/L)	1	0.97–1.02	0.787			
Neutrophil(10^9^ /L)	1.03	0.99–1.06	0.099			
Platelet(10^9^ /L)	0.99	0.99–1	0.286			
DD, *n* (%)	1.99	0.85–4.68	0.113			
INR, Mean ± SD	14.25	2.23–90.96	0.005	2.1	0.2–22.4	0.54
FIB(g/L)	0.43	0.23–0.8	0.007	0.44	0.2–0.97	0.042
ALB(g/L)	0.99	0.97–1.02	0.555			
BUN(mmol/L)	1.32	0.95–1.83	0.1			
Cr(µmol/L)	1.01	0.98–1.05	0.338			
CKMB(U/L)	1.04	0.95–1.14	0.432			
cTnT(ng/L)	1.03	0.91–1.17	0.65			

### Correlation of the trajectory of WBC count with treatment complications and transfusion support

No statistically significant differences are observed in the incidence of infection or liver dysfunction among the groups during induction. However, Group 1 is found to require significantly more intensive transfusion support during induction, including red blood cells, platelets, and plasma, with particularly high demands for red blood cell and platelet transfusions (*p* < 0.001), as reported in Table 4.

**Table 4:** Differences in the incidence of treatment-related complications and transfusion support among different groups.

Variables	Overall (*n* = 93)	Class 1 (*n* = 7)	Class 2 (*n* = 20)	Class3 (*n* = 53)	Class 4 (*n* = 13)	*p*-value
Infection, *n* (%)	76 (81.7)	6 (85.7)	18 (90)	42 (79.2)	10 (76.9)	0.801
Abnormal liver function, *n* (%)	19 (20.4)	2 (28.6)	5 (25)	11 (20.8)	1 (7.7)	0.606
Transfusion of plasma, Median (IQR)	200.0 (0, 900)	1,200.0 (400, 2,400)	800.0 (200, 1,450)	0.0 (0, 750)	200.0 (0, 800)	0.013
Transfusion of red blood cells, Median (IQR)	2.0 (0, 4)	4.0 (2.5, 7)	3.0 (2, 6)	2.0 (0, 2)	2.0 (2, 5)	<0.001
Transfusion of platelets, Median (IQR)	40.0 (20, 60)	80.0 (50, 100)	50.0 (40, 60)	20.0 (10, 40)	60.0 (40, 75)	<0.001

## Discussion

WBC count plays a critical role in the diagnosis and risk stratification of APL and is closely associated with the development of DS (16). Previous studies have demonstrated this association: Gao et al. (12), using GLMM, identify WBC doubling and WBC peak as independent risk factors for DS, while Laura Cicconi et al. (1), through multicenter data, show that elevated WBC count is a strong predictor of DS. As a dynamic and continuous parameter, WBC count is analyzed in this study using LGMM to investigate its trajectory over the first seven days of ATRA-ATO induction therapy in newly diagnosed APL patients and its association with DS. Four distinct trajectory classes are identified: Class 1 is characterized by the highest initial WBC count, which rises before declining, presenting an inverted V-shaped pattern; Class 2 shows a high initial WBC count that gradually decreases; Class 3 maintains consistently low WBC counts; Class 4 exhibits low initial WBC counts that gradually increase. Significant differences in clinical characteristics and DS risk are observed among these subgroups. Multivariable logistic regression analysis reveals that compared to Class 3 patients, those in Classes 1 and 4 have a significantly higher risk of developing DS. Additionally, patients in Class 1 require more frequent transfusion support during induction therapy.

In this study, nearly half of the patients receiving ATRA-ATO therapy develop leukocytosis during induction, which is consistent with previous findings reporting that up to 60% of patients treated with ATRA-ATO experience elevated WBC counts during induction (5, 17).

The results indicate that patients in Class 1 exhibit a higher risk of developing DS compared to those in Class 3, which aligns with prior studies demonstrating that high WBC counts at the initiation of therapy are an independent risk factor for DS (18), and that patients with continuously increasing WBC counts have an elevated risk of DS (1).

However, based on previous findings, the use of LGMM in this study provides a more nuanced classification of WBC trajectories, revealing that patients in Class 4 (low-level increasing group) also have an increased risk of DS compared to Class 3 (persistently low-level group), while those in Class 2 (high-level decreasing group) do not show a statistically significant difference in DS risk relative to Class 3 (persistently low-level group).Patients who begin with a low WBC count but show a sustained upward trend still face a markedly increased risk of differentiation syndrome. This finding underscores the limitations of relying on a single, static WBC threshold at treatment initiation for risk stratification. Consequently, during the first week of therapy, we should monitor dynamic WBC trends in all patients—not just those with high initial values—and consider early intervention (e.g., prophylactic corticosteroids) for anyone exhibiting a steady rise, regardless of their starting WBC level.On the other hand, the findings also extend prior knowledge regarding patients with high initial WBC counts. Even when the initial WBC count exceeds 10 × 10^9^ /L, different trajectory patterns during early induction therapy suggest varying DS risks. If the WBC trajectory demonstrates a continuous decline, the risk of DS is not significantly higher than that in patients with persistently low WBC counts. This indicates that early interventions leading to a declining WBC trajectory may reduce DS risk, even in patients presenting with high baseline WBC counts.

Therefore, these findings provide evidence supporting the NCCN guidelines, which recommend that a WBC peak exceeding 10 × 10^9^ /L serves as a clear indication for prophylactic corticosteroid use.

As for the mechanism underlying leukocytosis, several hypotheses have been proposed (17, 19). The proliferation hypothesis suggests that ATRA-ATO therapy targets the PML-RARα fusion protein to induce differentiation and maturation of APL cells; however, the differentiated granulocytes retain proliferative capacity, and an increased number of divisions during differentiation results in a rapid surge in mature granulocyte counts within a short period.

The cytokine storm hypothesis proposes that ATRA treatment significantly upregulates granulocyte colony-stimulating factor (G-CSF) expression, which reverses the antiproliferative effects of ATRA on HL-60 cells and alters the dynamics of c-myc gene expression, thereby promoting cell cycle progression.

In addition, existing preclinical studies suggest a potential link between leukocytosis during APL treatment and the development of DS. ATRA and ATO degrade the PML-RAR*α* fusion protein, releasing transcriptional repression, which in turn leads to abnormal proliferation and differentiation of promyelocytes, causing a rapid increase in peripheral WBC counts (18–21).

Inflammatory cytokines released by activated granulocytes, such as IL-1β, IL-6, and TNF-α, as well as vascular endothelial growth factor (VEGF), act in a paracrine manner to increase vascular permeability, promoting capillary leakage and organ edema (22). Furthermore, interactions between WBCs and endothelial cells are enhanced, and the upregulation of adhesion molecules, such as ICAM-1 and VCAM-1, contributes to microcirculatory disturbances, exacerbating tissue hypoxia and inflammatory responses (23, 24).

This study, through the application of LGMM, reveals for the first time a potential association between WBC trajectory patterns during ATRA-ATO therapy and the risk of DS in pediatric APL patients, which may provide a basis for early risk stratification. The findings are rigorously adjusted for multiple covariates and comprehensively assess secondary outcomes such as transfusion requirements, thereby enhancing their clinical translational value.

However, several limitations should be acknowledged. First, the single-center retrospective design may introduce selection bias, limiting the generalizability of the conclusions. Second, although Class 1 exhibits an extremely high incidence of DS, its small sample size limits the precision of the estimate; confirmation in larger, prospective cohorts is therefore essential. Third, unmeasured confounders, such as genetic variations or cytokine profiles, may influence WBC dynamics and the underlying mechanisms of DS development. Four, although the association between WBC trajectories and transfusion requirements is intriguing, it remains unadjusted for baseline coagulopathy and other confounders because of sample-size constraints; this relationship should be rigorously tested in larger, prospective cohorts. Additionally, although the seven-day observation window has practical clinical relevance, it may fail to capture later fluctuations in WBC counts.

## Conclusion

The trajectory of WBC counts during ATRA-ATO therapy in pediatric APL patients may serve as an indicator for predicting the risk of DS. Both the high-level increasing group and the low-level increasing group are identified as independent risk factors for DS, and patients in the high-level increasing group tend to require more frequent transfusion support during induction therapy.

## Data Availability

The raw data supporting the conclusions of this article will be made available by the authors, without undue reservation.
